# Intra pancreatic accessory spleen mimicking pancreatic insulinoma: a case report

**DOI:** 10.1093/jscr/rjaf138

**Published:** 2025-03-18

**Authors:** Fatima Chait, Kaoutar Imrani, Nourrelhouda Bahlouli, Ihssane Lahlou, Nabil Moatassimbillah, Ittimade Nassar

**Affiliations:** Radiology Department, Ibn Sina University Hospital, Mohamed V University Rabat, Morocco; Radiology Department, Ibn Sina University Hospital, Mohamed V University Rabat, Morocco; Radiology Department, Ibn Sina University Hospital, Mohamed V University Rabat, Morocco; Radiology Department, Ibn Sina University Hospital, Mohamed V University Rabat, Morocco; Radiology Department, Ibn Sina University Hospital, Mohamed V University Rabat, Morocco; Radiology Department, Ibn Sina University Hospital, Mohamed V University Rabat, Morocco

**Keywords:** accessory spleen, pancreatic, insulinoma

## Abstract

Accessory spleens are a congenital anomaly caused by abnormal fusion during embryonic development, leading to the presence of splenic tissue in unusual locations, such as the tail of the pancreas. Their pronounced arterial enhancement can cause them to be mistaken for a neuroendocrine tumor, particularly an insulinoma. We report the case of a 61-year-old patient with recurrent hypoglycemia who was referred for suspected neuroendocrine tumor.

## Introduction

An accessory spleen is a congenital anomaly found in about 10% of the population, with 16% of cases occurring in the pancreatic tail. While typically benign, distinguishing an intrapancreatic accessory spleen from a pancreatic insulinoma can be challenging. This paper presents the case of a 61-year-old woman with recurrent hypoglycemia, where imaging [computed tomography (CT) and magnetic resonance imaging (MRI)] revealed a highly vascularized mass in the pancreatic tail, resembling an insulinoma [1].

## Case report

We report the case of a 61-year-old hypertensive patient with no history of hypoglycemic medication use who experienced non-comatose hypoglycemia for a month, relieved by glucose administration. Physical examination and routine lab tests were unremarkable, but prolonged fasting led to a blood glucose drop below 50 mg/dl, causing sweating and mental confusion. Given the symptoms, a neuroendocrine tumor was suspected. Endoscopic ultrasound revealed a nodular lesion near the pancreatic tail, suggestive of an insulinoma, prompting further imaging for characterization.

**Figure 1:**
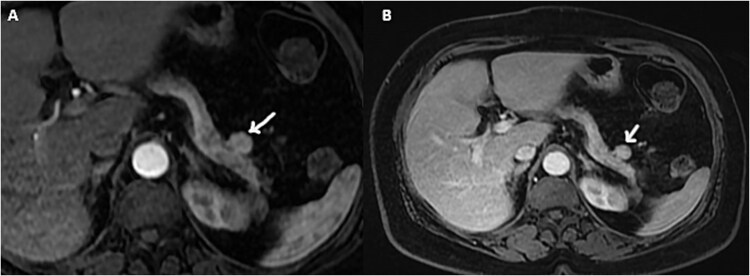
Axial gadolinium contrast T1 weighted images arterial (A) and portal phase (B) showing a small mass adjacent to the tail of the pancreas with intense arterial enhancement.

The pancreatic MRI confirmed the presence of a small, well-defined, round mass with regular contours, solid with an intermediate T2 and T1 signal, unrestricted diffusion, and appearing to be connected to the tail of the pancreas. Furthermore, the mass showed intense enhancement during the arterial phase and homogenization at portal phase (Fig. 1).

**Figure 2:**
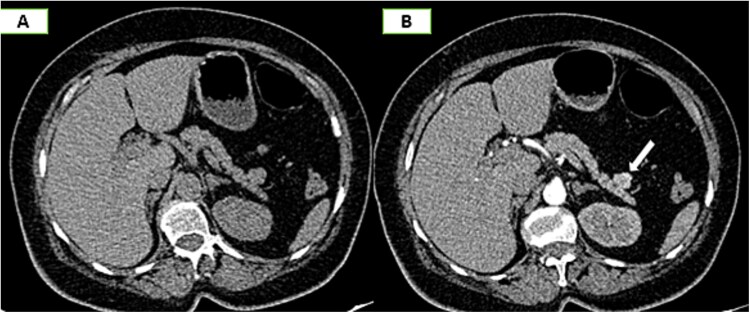
Axial sections of an abdominal non-contrast CT scan (A) and in the arterial phase (B) reveal a nodule adjacent to the tail of the pancreas that is hypervascular on arterial phase and follows the same pattern of enhancement as the spleen.

**Figure 3:**
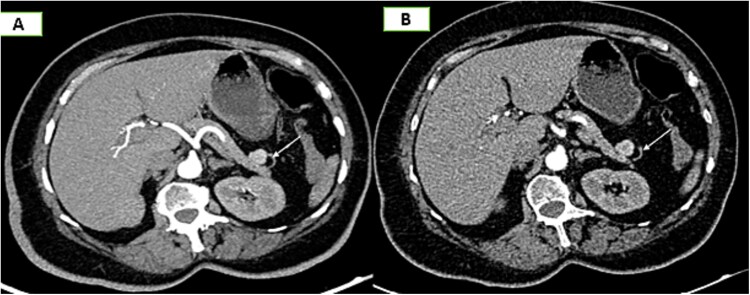
Axial sections of an abdominal CT scan in the arterial phase (A) and vascular reconstructions (B) demonstrate the vascularization of the accessory spleen through its pedicle.

On the complementary CT scan, this lesion exhibits the same tissue characteristics and enhancement as the spleen (Fig. 2), along with its own vascularization through a distinct vascular pedicle (Fig. 3), which is more suggestive ‘of an accessory spleen than an insulinoma of the tail of the pancreas.’

The patient's condition improved after the dietary errors were identified and rectified.

## Discussion

Accessory spleen is a congenital anomaly of splenic tissue with morphological and histological characteristics resembling a normal spleen, are discovered in up to 30% of unselected autopsy cases [2]. These can vary in size from a few millimeters to several centimeters, and they may occur as single or multiple entities. They are commonly situated near the splenic hilum, with Halpert and Gyorkey [2] reporting that one in every six accessory spleens is located in the pancreatic tail.

The spleen develops from several smaller components during embryogenesis, and the failure of these components to fuse can result in the persistence of one or more separate nodules. Each of these components is located outside the peritoneal cavity. It's important not to mistake them for splenosis, which is an acquired condition occurring inside the peritoneal cavity [3].

The most frequent locations are: splenic hilum (most popular) [4], gastrosplenic and splenorenal ligament, pancreatic tail, gastrocolic omentum, small bowel mesentery, stomach or bowel wall, and less often scrotum [5].

Conversely, intrapancreatic accessory spleen refers to an accessory spleen positioned either within the pancreatic tissue or its vicinity. It is a benign condition that often remains asymptomatic and is typically discovered incidentally through imaging studies. However, its discovery can provoke concerns regarding potential malignancy, as its radiological appearance can closely resemble that of highly vascularized pancreatic neuroendocrine tumors [6].

### Abnormal localization of splenic tissue can occur in various conditions

Polysplenia is a congenital syndrome characterized by multiple splenic nodules (2–16) in either upper quadrant, often associated with cardiovascular and pulmonary abnormalities [7].

Splenosis results from the implantation of splenic tissue after trauma or surgery, relying on neovascularization for blood supply, unlike embryologically derived accessory spleens [5].

Splenic clefts and lobulations arise from incomplete fusion of splenic tissue during development.

Imaging plays a major role in distinguishing accessory spleens within the pancreas from other pancreatic lesions; Baugh *et al.* emphasized the importance of employing CT scans since it is crucial to assess all pancreatic masses using a triphasic CT approach. In the case of an intra-pancreatic accessory spleen (IPAS), it typically appears as a clearly defined mass characterized by heterogeneous enhancement or a distinctive zebra-stripe pattern during the initial phase. This pattern arises from variations in blood flow between the red and white regions [8]. Bhutiani *et al*. also confirm the role of CT in distinguishing intra- or pre-pancreatic accessory spleens [9].

Nuclear medicine is essential for assessing accessory splenic tissue and distinguishing it from pathological conditions like lymphadenopathy or tumors. The most sensitive imaging methods are Tc-99 m sulfur colloid and Tc-99 m heat-denatured RBC scans.

Tc-99 m Sulfur Colloid Scan: highlights splenic tissue due to its distinct uptake pattern, useful in detecting residual spleen after splenectomy.

Tc-99 m Heat-Denatured RBC Scan: the most specific test, as splenic tissue selectively sequesters denatured RBCs, ensuring clear visualization, especially when sulfur colloid imaging is inconclusive [9].

In our patient, the CT scan played an important role in characterizing the lesion located in the tail of the pancreas, supported by the following arguments:

Arterial enhancement within the lesion, resembling that of the spleen and reminiscent of the zebra-stripe pattern.

The identification of a vascular pedicle supplying the accessory spleen (Fig. 3).

This allowed us to make the diagnosis of an accessory spleen adjacent to the tail of the pancreas instead of that of an insulinoma.

The main differential diagnoses of IPAS include hypervascular pancreatic masses such as neuroendocrine tumors, solid pseudopapillary tumors, and hypervascular metastases.

Neuroendocrine tumors appear as small, highly vascular lesions on arterial-phase CT and MRI. Non-functional tumors tend to be larger with cystic necrosis, while IPAS presents as a compact, uniform nodule.

Solid pseudopapillary tumors are typically found in women aged 20–40 and appear as well-defined masses over 5 cm. On non-contrast CT or MRI, they show heterogeneous density, with solid components exhibiting subtle peripheral enhancement, usually lower than that of the pancreatic parenchyma.

Pancreatic metastases commonly arise from primary malignant tumors such as lung cancer, breast cancer, gastrointestinal tumors, and renal tumors [10].

## Conclusion

In conclusion, accessory spleens are common and have distinctive CT features. They appear as well-defined, circular masses with homogeneous enhancement, usually under 2 cm. Their presence near the splenic hilum and pancreatic tail can make differentiation from neuroendocrine tumors challenging.
